# Benefits for Exempt Persons

**Published:** 1914-02-28

**Authors:** 


### NATIONAL INSURANCE ACT DEVELOPMENTS.

### Benefits for Exempt Persons.

Persons who have been exempted from the payment of contributions under the National Insurance Acts, but in respect of whom contributions have been paid by their employers, are now entitled, by virtue of the provisions of the Act of 1913, to certain benefits, subject to regulations made by the Insurance Commissioners. These regulations have now been issued, and as the matter is one of considerable importance, as well as of general interest, we propose to indicate the nature of the benefits and the conditions that must be fulfilled before they can be enjoyed. It will be remembered that, although the Act of 1911 imposed insurance compulsorily upon all those coming within the definition of an "employed person," yet, by a special provision, permission was given whereby those who fulfilled the necessary conditions might escape their obligation to pay the contributions. The employer of such a person was not, however, entitled to reap any advantage by the employment of an exempted person, and had to pay the employer's share of the contribution just as though his employee had been fully insured.

In consideration of the fact that the worker did not pay any contribution himself no benefit whatever was to be derived by him under the Act, and thus the position was that, although contributions were being paid on his behalf by his employer, there was nothing to determine what should be done with those contributions beyond the direction in the Act that "they shall be carried to such an account and dealt with in such manner as may be prescribed by regulations made by the Insurance Commissioners." By this simple artifice the ultimate disposal of the fund that would necessarily accumulate from these contributions was shelved until a convenient opportunity arose for it to be dealt with. That opportunity came when the Amendment Bill was before the House last year, and the result was, as already mentioned, that the exempt persons are themselves to benefit from the contributions paid in respect of them.

Whether this method of dealing with the matter was wise or otherwise remains to be seen, but it would certainly seem that some other solution will ultimately have to be found.

### Qualifications for Exemption.

There are three classes of persons who, although employed within the meaning of the Acts, are entitled to claim exemption from the payment of contributions. The first two were provided for in the principal Act, while the third was added by Section 5 of the Amendment Act. The qualifications are as follows: The person must be (*a*) in receipt of a pension or income of the annual value of £26 or upwards, *not dependent upon his personal exertions*; or (*b*) ordinarily and mainly dependent for his livelihood upon some other person; or (*c*) ordinarily and mainly dependent for his livelihood on the earnings derived by him from an occupation which is not employment within the meaning of the Act.

It will at once be seen that the intention was to relax the compulsory character of the Act in favour of those who, when they fell ill, would be provided for apart from any benefit they would obtain under the Act. Two things should be noted in connection with the provisions for exemption; first, that no obligation was placed upon any person who might be entitled to claim the advantages to make him avail himself of them—he was entirely free to exercise the option conferred upon him as he might choose; and, secondly, that, in order to obtain the privilege of exemption, it was made necessary to obtain an exemption certificate, and evidence had to be produced to the satisfaction of the Commissioners.

Some confusion has arisen on several occasions between the description here given of "exempt persons" and those engaged in an "excepted employment." Neither class are insured persons, but, while no contributions whatever are payable either by the employer or by the worker who is engaged in an excepted employment, in the case of exempt persons the employer has to pay his share precisely as if the person were insured.

### The Benefit for Exempt Persons.

The Act of 1913 did not leave the disposition of the funds derived from the employers' contributions entirely at the discretion of the Insurance Commissioners, for while it provided that the funds were to be administered in accordance with regulations made by them, it directed that medical and sanatorium benefits were to be conferred on persons holding certificates of exemption. It will, of course, be noted in passing that these are the benefits administered by the Insurance Committees and not by approved societies. The regulations, which have now been issued, set forth in detail the conditions under which these two benefits may be claimed. The regulations are typically complex, and must be very carefully studied to appreciate all the safeguards that they are designed to create for the protection of the fund against fraud, and it would be useless to attempt any detailed description of them here. Briefly stated, however, they require that at least twenty contributions must have been paid in respect of an exempt person before he is entitled to any benefit. When twenty contributions have been paid he may apply to the Commissioners, and, after the lapse of six weeks from the receipt by them of the application, he will become entitled to the benefits subject to the provisions of the Acts and of any regulations made thereunder. Failing any such application to the Commissioners the exempt person will automatically come into the right to obtain the benefits at the commencement of the next "benefit period"—that is, the six months from April 1 or October 1—following the "contribution period"—that is, the six months for which the contribution cards are current—in which the payment of twenty contributions was completed. The right to continue to be entitled to the benefits depends upon the payment of the requisite number of contributions. These must not be less than thirteen in any half-yearly "contribution period," but, in reckoning the number of contributions, should they fall short of thirteen, allowance by way of credit will be given for any excess over that number that have been made in the two preceding "contribution periods."

If after allowing for any such credits from former periods the number of contributions is still less than thirteen, the person will be suspended from benefits as from the commencement of the "benefit period" next ensuing.

Exempt persons, in respect of whom on the attainment of the age of seventy no further contributions are to be paid, will be entitled to the two benefits for the remainder of life, provided that on the attainment of that age at least twenty-seven contributions have been paid on their behalf. This right is, however, subject to certain qualifications depending upon the previous suspension of the person from benefit, the details of which it is not necessary to mention. The general effect of the regulations on this point, then, is that an exempt person must be employed within the meaning of the Act for at least thirteen weeks each half-year in order that he may be continuously entitled to the benefits provided.

### The Administration of the Benefits.

The benefits are to be administered in the same way as medical and sanatorium benefits are dealt with in the case of ordinary insured persons. Thus the Insurance Committees will furnish medical cards to all exempt persons entitled to benefit, and it is to these bodies that exempt persons will apply if they desire to receive sanatorium treatment. There is, however, one slight modification in regard to the administration of the medical benefit that merits particular notice. If the total income of an exempt person from all sources exceeds £160 a year, such person is *required* by virtue of Section 9 of the Act of 1913 to make his own arrangements for receiving medical treatment and attendance. A contribution will, of course, be made by the Insurance Committee towards the cost of the treatment equivalent to the sum that would have been paid if his income had not exceeded £160 a year. But the point is that the arrangements will not be "panel" arrangements, but will be a matter to be decided between the individual person and the practitioner he selects to attend him. This is obviously just, for it may well happen that an exempt person has an income much in excess of £160 a year. If, for instance, he is only engaged in employment within the meaning of the Act at a *rate* less than £160 a year for one day a week, contributions will have to be paid by his employer, but he may have an assured income of £500, or by his normal occupation earn an even larger sum. He would still be entitled to claim the benefits of an exempt person, if he obtains an exemption certificate, and it would be manifestly inappropriate that any doctor should be compelled to accept such a patient on "panel practice" terms.

### Is the New Arrangement Desirable?

Having given a description in outline of the new scheme for benefiting exempt persons, we ask the pertinent question whether it is desirable in principle that these benefits should have been granted. To answer that question we would first draw attention to the fact that before a person can obtain a certificate of exemption he must produce evidence to show that he has means available for his support in time of illness. Furthermore, it is *prima facie* evidence that he does not desire to be insured that he takes the trouble to obtain and renew from time to time such certificate. There is, therefore, no plea on the ground of the necessity of such persons to provide them with any benefit whatever. Moreover, they are not required to pay any contribution, and the benefit is in consequence a free gift, and, like many other gifts at the expense of the nation, will probably not be appreciated by those to whom they might possibly be of service, but will hold out a tempting bait, in spite of all precautions, to those who are on the alert to take advantage of their fellows.

The new scheme is, in our opinion, more doubtful still, for, on the evidence of the actuarial report furnished to Parliament when the Bill was being considered, it is estimated that the benefits will cost the State £19,000 a year over and above the contributions that are paid by the employers. It is true that this is not a large sum for a wealthy nation to find, but, nevertheless, it is wasteful extravagance if it is only to benefit comparatively few—the estimated number is only about 70,000—and those few neither require the subvention nor have any logical claim to it.

The actual destination of the fund—apart from that applicable to sanatorium benefit—is the medical benefit funds of the Insurance Committees, and the money will thus further swell the remuneration of the "panel practitioners." Now if our hypothesis is correct that a large proportion of the exempt persons will not avail themselves of the benefit, we are faced with the fact that the "panel" doctors will be remunerated, on the distribution of funds, for many more unallotted persons than at present, or, in other words, for work not done.

There is only one solution to the difficulty, in our opinion, and that is to repeal the section of the Act which requires contributions to be paid by employers in respect of exempt persons. The only semblance of justification for requiring the payment to be made is that, unless the employer has to pay his own contribution, the exempt person will be sought after to the detriment of the ordinary insured person. But threepence a week is not sufficient inducement to any employer to discharge an insured workman, and seek an exempt workman, nor to make any difference in his choice between two applicants otherwise equally fit for a vacant post.

The repeal would not only save the employers and the State unnecessary expenditure directly incurred, but would obviate the cost of administering such complicated regulations.

